# Formation and Applications of the Secondary Fiber Bragg Grating †

**DOI:** 10.3390/s17020398

**Published:** 2017-02-18

**Authors:** Bai-Ou Guan, Yang Ran, Fu-Rong Feng, Long Jin

**Affiliations:** Guangdong Provincial Key Laboratory of Optical Fiber Sensing and Communications, Institute of Photonics Technology, Jinan University, Guangzhou 510632, China; rayiori@163.com (Y.R.); ffrhust@126.com (F.-R.F.); iptjinlong@gmail.com (L.J.)

**Keywords:** fiber Bragg grating, photosensitivity, Talbot effect, distributed Bragg reflector fiber laser, high temperature resistance

## Abstract

Being one of the most proven fiber optic devices, the fiber Bragg grating has developed continually to extend its applications, particularly in extreme environments. Accompanying the growth of Type-IIa Bragg gratings in some active fibers, a new resonance appears at the shorter wavelength. This new type of grating was named “secondary Bragg grating” (SBG). This paper describes the formation and applications of the SBGs. The formation of the SBG is attributed to the intracore Talbot-type-fringes as a result of multi-order diffractions of the inscribing beams. The SBG presents a variety of interesting characteristics, including dip merge, high-temperature resistance, distinct temperature response, and the strong higher-order harmonic reflection. These features enable its promising applications in fiber lasers and fiber sensing technology.

## 1. Introduction

Fiber Bragg gratings (FBG) are one of the most well-known optical fiber devices due to their compact size, narrow bandwidth, immunity to electromagnetic interference, and their inherent multiplexing capability [1]. FBGs have been exploited as distributed reflectors in fiber lasers, spectral filters for wavelength-division-multiplexing (WDM) applications, microwave photonic signal processing, and dispersion compensators in optical telecommunications. They are also important photonic sensors, which are capable of measuring temperature [2], strain [3], bending [4], vibration [5], acoustic waves [6], refractive index [7] and, recently, even biomolecules [8]. By assembling and building a wavelength- and/or space-division-multiplexed FBG array, one can monitor the security status of large infrastructures and green energy facilities. The FBG technology has been fast growing in recent years, improving the FBG’s performances in tunability, thermal resistance, and lifetime.

A variety of different FBGs have been fabricated with different mechanisms of grating formation [9,10]. They have exhibited their own characteristics which enable various applications in different conditions. Type-I FBGs, which are known as common gratings, have positive index modulations and average index changes. The grating formation arises from the changes of the defect polarizability, densification, and stresses. They can stand temperatures below 350 °C.

Type-Ia FBGs are typically formed in hydrogen-loaded fibers. The grating inscription would consume a large amount of cumulative fluence, enabling a greater bulk average index change. A larger red-shift of the Bragg wavelength would occur during inscription. The swelling by hydrogen provides the stress relief through the anisotropic OH formation. The Type-Ia FBG can withstand the temperature up to 500 °C.

Regenerated FBG is usually fabricated by post-annealing an over-saturated Type-I FBG written in a heavily hydrogen-loaded fiber. After the full decay of the Type-I seed FBG, a new FBG would regenerate at high temperature, presenting outstanding thermal resistance despite the weak grating strength. The regenerated FBGs have been tested at extremely high temperatures (over 1100 °C) with good thermal stability. The grating regeneration is probably attributed to the change of the chemical component of hydroxyl ions at ultra-high temperature.

Type-II FBG is usually fabricated via the ablation of a high-intensity laser beam exceeding the damage threshold of silica. The physical damage-based periodical structure in the silica fiber enhances the thermal stabilization. The Type-II FBG can survive at a temperature of 1000 °C.

The formation of a Type-IIa FBG imparts high demands on the host fiber. The fiber should have a small core diameter, high photosensitivity, and free of hydrogen loading. The Type-IIa FBG would experience a rollover evolution following a saturated Type-I grating. The regrowth resonance presents an obvious blueshift in the reflected wavelength during post-inscription, as a result of the stress relief by the anisotropic relaxation through the dilation of the core-network. Therefore, it is also denoted as “negative-index grating” or “Type-In grating”. The Type-IIa FBG can work at temperatures up to 800 °C. It has attracted increasing attention since a new mechanism in photosensitivity needs to be clarified for the negative index changes.

In recent years, Type-IIa FBGs written in different fibers have been reported, including B/Ge (Sn/Ge) co-doped fibers, high Ge-doped fibers, photonic crystal fiber, and microfibers [11,12,13,14,15,16,17,18,19,20,21,22]. These studies focused on passive fiber-based Type-IIa FBGs. Most recently, we had put forward the study on the Type-IIa FBG directly written in rare-earth doped fibers [23]. The high inscription efficiency endorses the active fiber-based Type-IIa FBG in acting as a strong Bragg reflector, naturally integrated in the laser cavity. More importantly, a new type of grating was discovered, accompanying the formation of the Type-IIa grating. The new type of grating presents a delayed growth and shorter wavelength than the grating which is initially designed. We name this new grating a “secondary Bragg grating” (SBG). In this article, we describe our recent works on SBGs, including the investigation of their formation mechanism, and the applications in high-temperature-resistant fiber grating lasers, thermally-triggered fiber grating lasers, and the higher harmonic grating fiber lasers.

## 2. Formation of the SBGs

**Figure 1:**
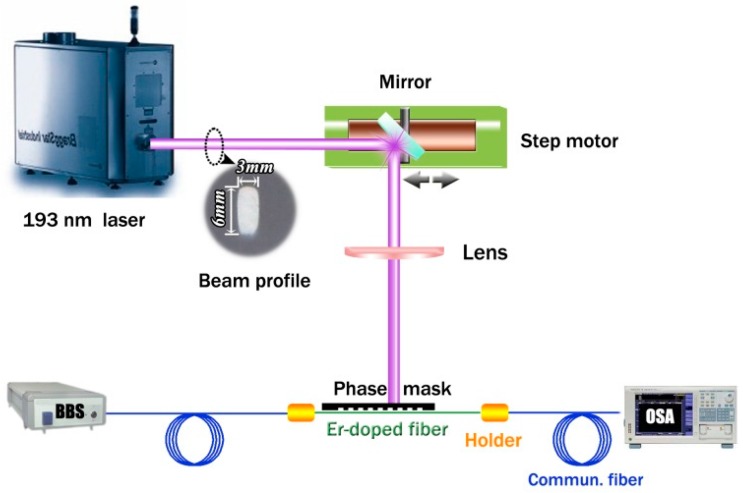
Experimental setup for FBG inscription; BBS: broadband source; OSA: optical spectrum analyzer.

The grating inscription setup is shown in Figure 1. A 193 nm ultraviolet excimer laser (BraggStar Industrial, Coherent. Inc., Santa Clara, CA, USA) is employed for grating inscription. A 120 mJ/cm^2^ laser density per pulse is incident upon the fiber after focusing via a cylindrical lens. The repetition rate of the laser is set to 200 Hz. The exposing length is 3 mm, which is determined by the laser beam profile. A standard phase mask with a pitch Λ_pm_ = 1067.17 nm is adopted. The diffraction efficiencies of the phase mask are ~39% at first, and ~2% at second orders, respectively. A broadband light source with a wavelength range of 1500–1600 nm, average power density of −15 dBm/nm, and an optical spectrum analyzer (OSA) with a resolution of 0.02 nm (AQ6370C, Yokogawa, Tokyo, Japan) are used to monitor the grating transmission spectrum.

The fiber used here is a commercial photosensitive Er-doped fiber. It has a cladding diameter of 125 μm and a core diameter of 3.0 μm. Due to the heavy germanium dopant in the fiber core, the fiber has a large numerical aperture (N.A.) of 0.24 and a high photosensitivity to UV light. It can meet the inscription requirements of Type-IIa Bragg gratings, considering the small core and high photosensitivity. The cut-off wavelength is 915 nm. The absorption to 980 nm pump light of the fiber is approximately 5 dB/m.

**Figure 2:**
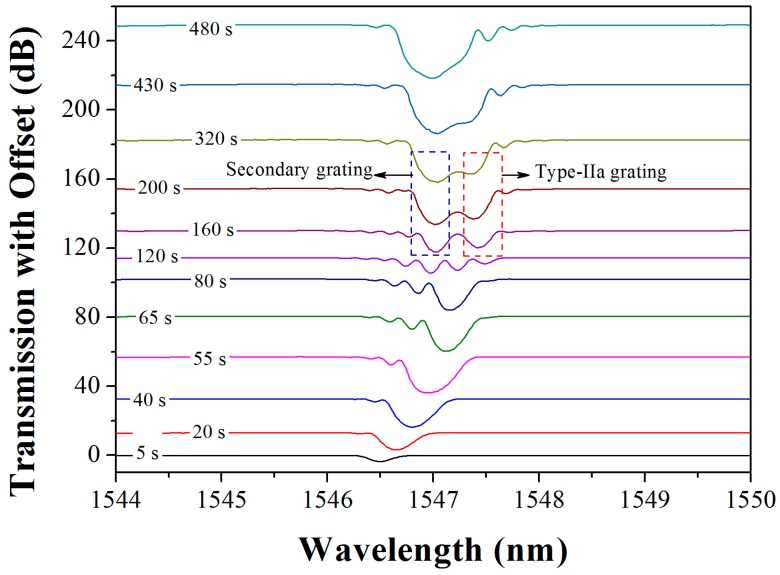
Evolution of the Type-IIa grating and the secondary grating.

The fiber is pre-loaded by a tension of 0.2 N to accelerate the formation of the Type-IIa grating. Figure 2 shows that the grating spectrum evolves with increasing cumulative UV fluence. It can be seen that the grating grows quickly at first, presenting the evolution of the Type-I grating. Only with 55 s exposure, the Type-I grating becomes saturated, presenting a reflectivity of ~20 dB. Then the grating starts to decay and eventually achieves a minimal strength of ~4 dB during another 65 s exposure. In the following exposing process, the original primary dip at a longer wavelength regrows with a blue wavelength shift, presenting the Type-IIa grating evolution. Finally, a Type-IIa grating with a reflectivity over 33 dB is obtained after another six minutes.

**Figure 3:**
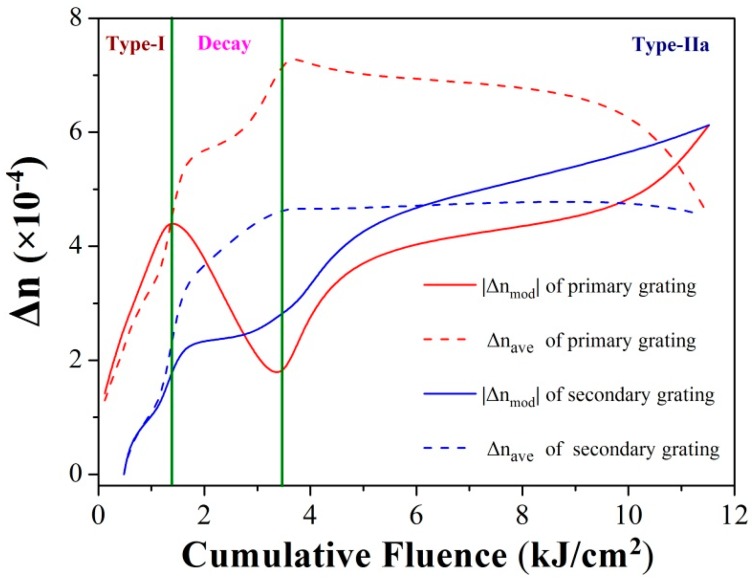
Variation of the indices throughout the grating inscription.

Figure 3 illustrates the overall refractive index change of the grating during the inscription. It can be clearly seen that the primary grating experiences a typical “roll-over” evolution process and presents a blue-wavelength-shift at the third stage, in accordance with the formation of the Type-IIa grating.

Here, an abnormal strong dip can be observed at a shorter wavelength with a reflectivity of 10 dB at the end of the grating decay stage. Moreover, it also grows during the Type-IIa stage and eventually contributes to the strength of the Type-IIa grating. According to the variation of the refractive index in Figure 3, the characteristics of this abnormal dip can be revealed. At first, it grows, following the primary dip in Type-I stage, with less index changes both in bulk average and modulation, exhibiting lesser strength at shorter wavelengths. Thus, in order to distinguish it from the primary Bragg grating, we name the dip as the “secondary Bragg grating”. Moreover, the secondary dip grows stronger, exceeding the primary dip, which attenuates in the decay stage. In the Type-IIa stage, the secondary dip continues growing, but maintains its wavelength. The Type-IIa dip, which presents a negative index change, approaches the secondary dip gradually. As a sequence, the two dips would integrate through a sufficient cumulative fluence, eventually.

The double-dip phenomenon in the grating is ascribed to the Talbot-type-fringes of the inscription UV laser. For a commercial phase mask, ±2 diffraction orders are not fully suppressed (~2% remaining in our experiments), affecting the interference fringes formed by the ±1 diffraction orders. As a result, Talbot-type fringes are formed in the transversal direction, presenting a sinusoidal variation. The period of the variation is defined as Talbot length. The interleaving transverse index variations with intervals of Λ_pm_/2 present π phase difference [17,24,25,26].

**Figure 4:**
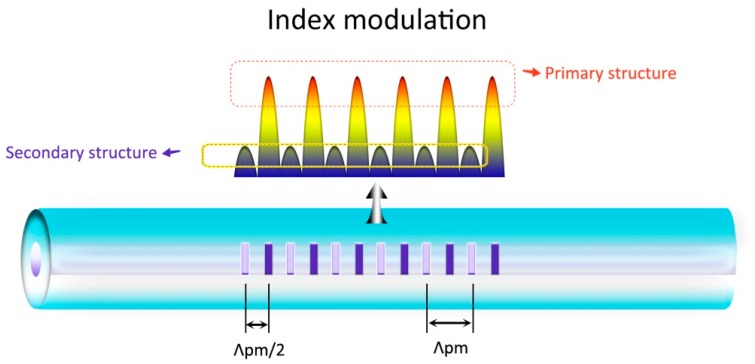
Schematic of the interleaving index-modulation-structures due to the Talbot-type-fringes.

In our experiment, the Talbot length is estimated to be 6.2 μm from the UV laser wavelength and the phase mask period. As the Talbot-type fringes are imprinted in fiber core, the sinusoidal index variation is also formed. Therefore, two second-order-diffraction modulation structures interleaving along the core axis act on the fiber core both with the period of Λ_pm_. By integrating the sinusoidal index variation, the overall effect of the index change (OEIC) can be obtained. Owing to the π phase difference of the index variation between those two structures, the OEIC contrast of the two structures is highly dependent to the core diameter. Considering that the diameter of the active fiber here is 3.0 μm, approximately just the half of the Talbot length, two modulation structures would exhibit significantly OEIC contrast, as shown in Figure 4. The formation of the secondary dip is attributed to the weak influence grating structure by a large amount of exposure of the Type-IIa grating formation, presenting the later growth and shorter wavelength than the primary dip, which is the outcome from the strong-influence grating structure.

## 3. Applications of the SBGs

The secondary Bragg grating presents a variety of interesting characteristics and shows promising potential in the area of the fiber lasers.

### 3.1. High-Temperature-Resistant Short-Cavity DBR Fiber Laser

It is well known that Type-IIa fiber Bragg gratings exhibit considerable resistance in high-temperature environments without thermal decay. Moreover, the secondary grating would contribute to the whole grating reflectivity as it is integrated with the Type-IIa grating. According to the Figure 2, 33 dB reflectivity of Type-IIa grating can be obtained by UV exposure within eight minutes. Therefore, we can fabricate a distributed Bragg reflector (DBR) fiber laser through directly inscribing a pair of Typy-IIa gratings into the active fiber as Bragg reflectors. The strong optical feedbacks of the Bragg reflectors can support the laser oscillation within an ultra-short laser cavity.

**Figure 5:**
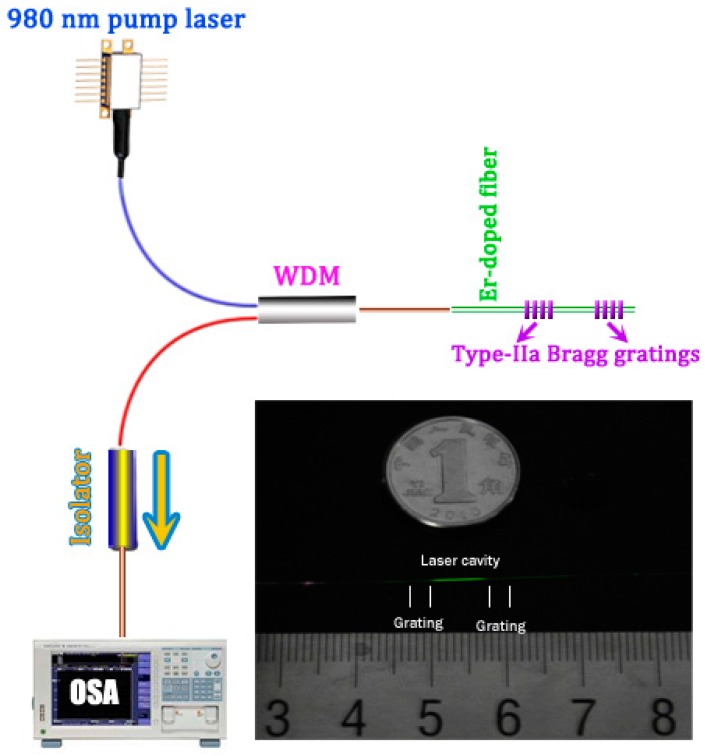
Schematic of the DBR laser structure. Inset: the photograph of the DBR laser. WDM: wavelength division multiplexer.

As shown in Figure 5, we fabricate a Type-IIa grating into the Er-doped fiber with 480 s exposure to produce a stronger reflector. Then the UV beam moves longitudinally by 10 mm. With another 400 s exposure at the new position, a weaker Type-IIa reflector is fabricated. The entire laser length is 13 mm, including the two Type-IIa gratings. The inset of Figure 5 shows the photograph of Type-IIa grating based DBR fiber laser. The Er-doped fiber emits green fluorescence when excited at 980 nm. The green fluorescence in the grating regions is much weaker than that in the intracavity region. It seems that the formation of Type-IIa grating inhibits the energy transfer upconversion in Er-doped fiber.

**Figure 6:**
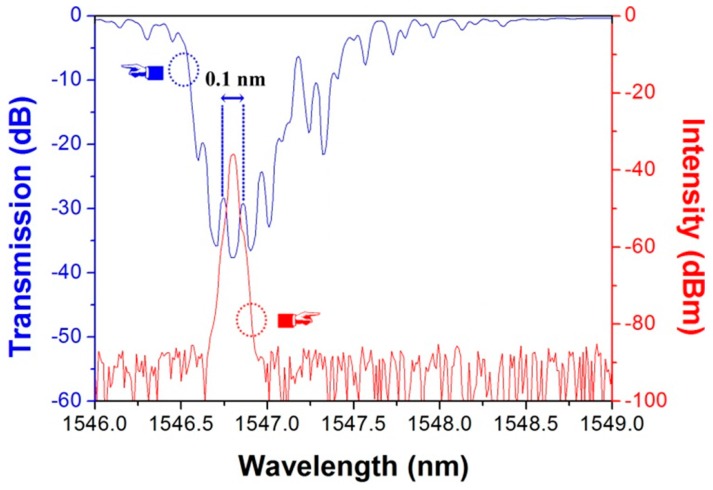
Spectra of the F-P interference and DBR laser output.

Figure 6 shows the transmission spectrum of the Fabry-Perot cavity and the laser output spectrum recorded by an OSA with resolution set to 0.02 nm. Owing to the short cavity length, the longitudinal mode space is 0.1 nm. The laser oscillates at 1546.8 nm at room temperature. In the experiments, the laser performs a stable single-longitudinal-mode operation, which is a very important quality for applications in sensing and communication systems.

**Figure 7:**
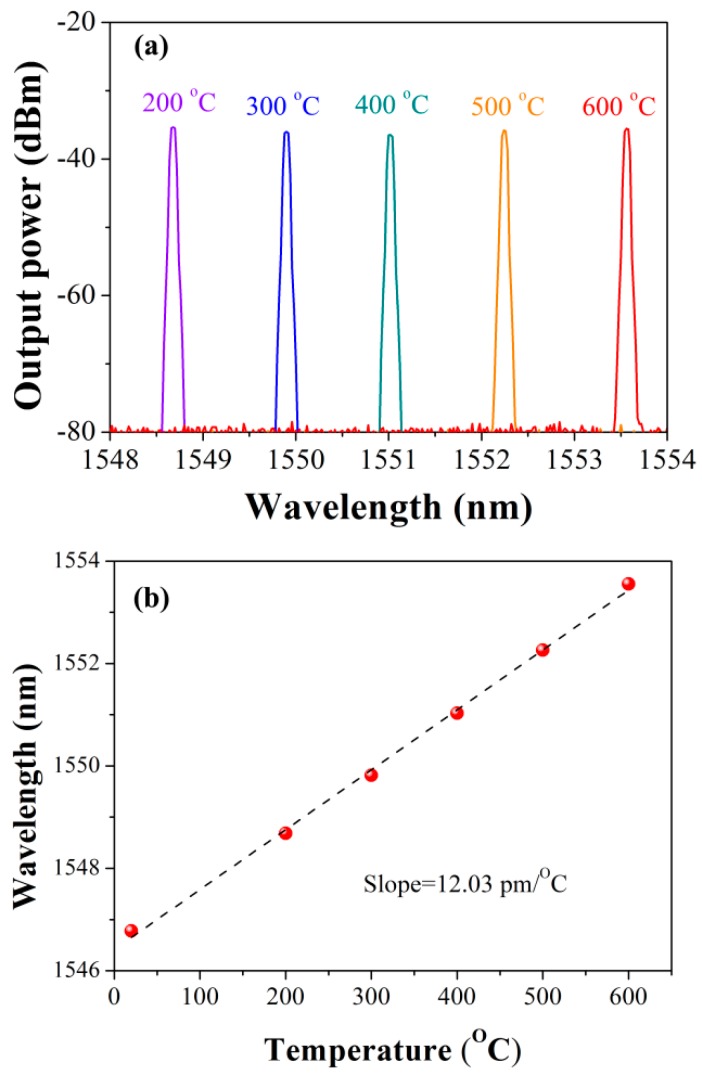
(**a**) Measured output spectra of the laser at different temperatures; and (**b**) lasing wavelength versus temperature.

The performance of the DBR laser at high temperature is tested. The laser cavity is placed in a tube oven which can be operated from room temperature to 1200 °C. We recorded the laser output spectrum with one hundred centigrade steps increasing from 200 °C to 600 °C. The duration under the temperatures below 600 °C are set to one hour after the stabilization. At 600 °C, we provide a longer time of 2 h. As is shown in Figure 7, the lasing wavelength redshifts along with the temperature enhancement, presenting a highly linear response. The sensitivity of the laser can be calculated as ~12 pm/°C.

**Figure 8:**
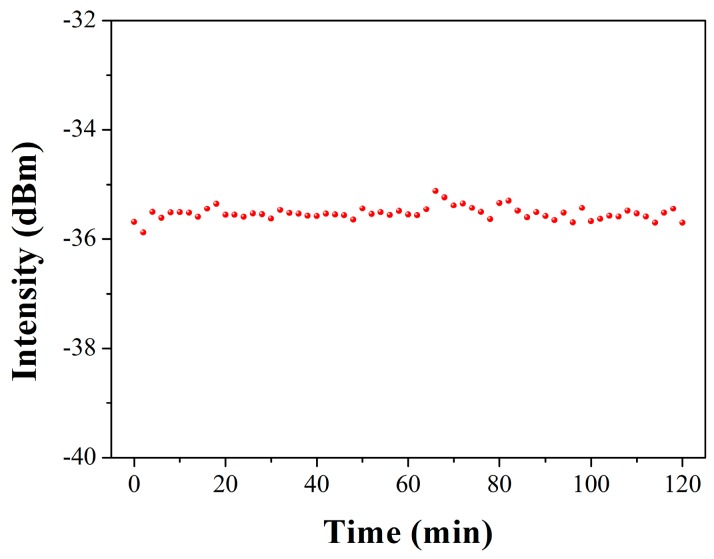
Stability test result of the laser at 600 °C for 2 h.

The stability of laser output power at high temperature of 600 °C is presented in Figure 8. The average power is −35.5 dBm with standard deviation of ~0.18. Therefore, it can be concluded that the Type-IIa grating based DBR fiber laser shows good resistance to 600 °C, enabling its potential of acting laser sensors applied in harsh environment and laser source with high stability, as well as a large dynamic tuning range of wavelengths [23].

### 3.2. Thermally-Triggered DBR Fiber Laser

If the UV exposure ends just before the grating integration, the two dips arising from the secondary grating and Type-IIa resonance remain separated.

**Figure 9:**
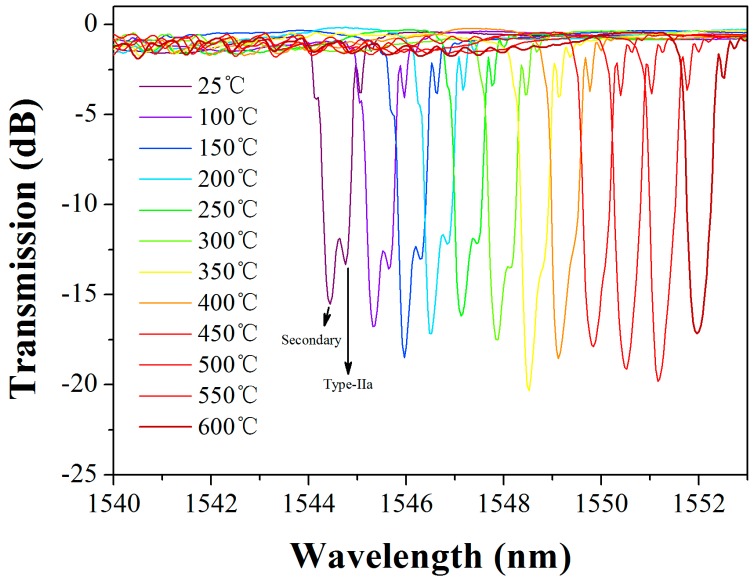
Measured transmission spectra of a two-dip grating at different temperatures.

Figure 9 shows the temperature response of a two-dip grating with an initial wavelength separation of 0.28 nm. The transmission spectrum of the grating is recorded at different temperatures from room temperature to 600 °C. The secondary resonance which locates at short wavelength side shows a higher temperature sensitivity than the Type-IIa resonance. Therefore, the wavelength separation decreases with temperature, and the two dips tend to merge with each other as temperature increases. When the temperature is increased to 500 °C, the two dips integrate entirely. As a result, the grating reflectivity is strongly enhanced due to the overlapping of the two grating components.

**Figure 10:**
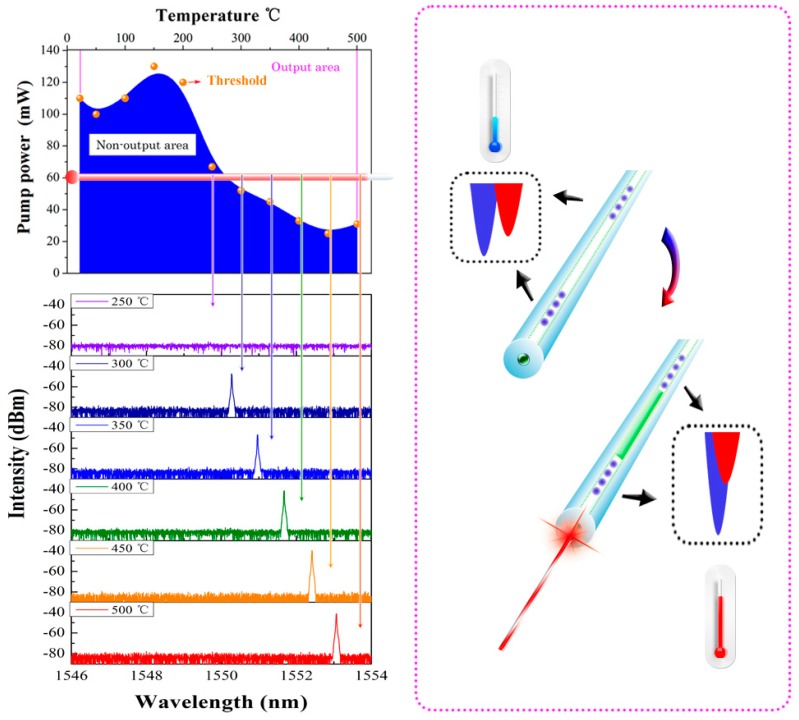
Pump threshold and the thermal trigger (**left**) of the two-dip grating based DBR fiber laser (**right**).

The characteristic of those dual-dip gratings provide the opportunity to form a DBR fiber laser whose threshold can be thermally tuned. A pair of such two-dip gratings is inscribed at both end of a piece of Er-doped fiber with certain spacing. The cavity length is 17 mm. A 980 nm pump laser with output power tunable from 0 to 500 mW is connected to the laser cavity. Thus, the thresholds of the DBR laser can be measured by tuning the pump power at different temperatures as shown in Figure 10.

It can be observed that the pump threshold varies with the temperature. As the temperature increases to 250 °C, the threshold is significantly reduced to less than 70 mW indicating that the secondary dip starts to overlap with the Type-IIa dip. Thus, higher reflectivity of the gratings are enabled. Higher temperature yields lower pump power for laser oscillation because the two dips are better overlapped. At 450 °C, the threshold reaches the minimum value, just a 30 mW pump power is enough to establish the laser output. Here, given that the intensity of the ultra-short DBR laser maintains during the temperature increasing which is shown in Figure 7a of Section 3.1, the variation of the laser threshold in this case is mainly attributed to the grating reflectivity enhancement.

Figure 10 shows two regions denoted as “Output area” and “Non-output area”, divided by the threshold curve. If the combination of pump power and temperature falls in the output area, the laser can be triggered. Otherwise, there is no laser output. However, if we set the combination in the non-output area, but close to the thresholds of the temperature above 150 °C, the laser can be triggered by increasing the temperature while the pump power is maintained. For example, as shown in Figure 10, as the pump power is set to 60 mW, the laser cannot be triggered if the temperature is below 250 °C. However, if we increase the temperature to 300 °C, the laser can be triggered. With the further increase of temperature, the laser power is accordingly enhanced due to the strengthening of the grating reflectivity. As the temperature increases to 450 °C, the highest power can be obtained, up to −40 dBm, probably caused by the annealing of the secondary dip as the temperature is higher than 450 °C. Nevertheless, depending on the high temperature resistance of Type-IIa grating, those thermally-triggered DBR fiber lasers can be potentially used as high-temperature alarms, of whose warning temperature can be flexibly managed through setting the pump power [27].

### 3.3. Third Harmonic Grating Fiber Laser Operating at 1-μm

The larger index change contrast of the two interleaving modulation structures which is essential in forming secondary grating can also lead to a high strength resonance at the third harmonic wavelength.

**Figure 11:**
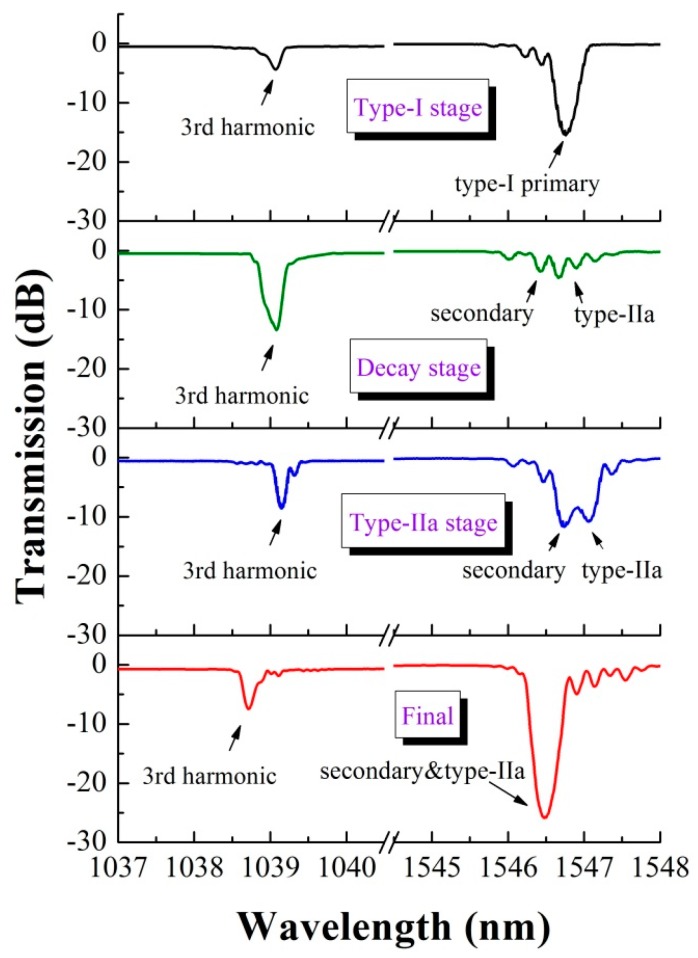
Spectral evolution of the “secondary-Type-IIa” grating at 1 μm and 1.5 μm bands.

The resonant wavelengths of the FBG can be expressed as [25,28] in Equation (1):

(1) $$\lambda \left(j\right)=\frac{2}{j}n_{eff}\Lambda$$

where *λ*(*j*) is the reflection wavelength at the harmonic orders *j* = 1, 2, 3, …, Λ denotes the grating period and *n_eff_* is the effective index. For an ideal phase mask, only the ±1 diffraction orders of the UV light exist, the Bragg wavelength *λ_B_* is the first harmonic reflection (*j* = 1) and Λ is half of the pitch of the phase mask (Λ_pm_). For practical cases, the participation of the ±2 diffraction orders results in Talbot-type fringes, two modulating structures which are both dominated by Λ = Λ_pm_ interleave into the fiber core. *λ_B_* is primarily attributed to the second harmonic resonance (*j* = 2). Therefore, it can be predicted that a strong dip would appear at the wavelength around 1 μm in accordance with the third harmonic resonance. We monitor the evolution of this secondary-Type-IIa grating in 1 μm and 1.5 μm wavelength-windows simultaneously, as shown in Figure 11. At the wavelength of ~1039 nm, a resonance dip can be observed in all of the growing stages of the secondary-Type-IIa grating. Note that there is only one dip other than the double dips at 2/3 *λ_B_* which was reported in [17] because only a half Talbot-layer is supported in the fiber core, avoiding the π-phase shift structures [29]. At the end of the primary grating decay stage, the 2/3 *λ_B_* resonance reaches the maximum strength with reflectivity higher than 95%, denoting the largest overall photorefractive effect contrast between the two modulating structures. Subsequently, the third-harmonic-dip decays gradually along with the integrating of the secondary and Type-IIa dips, unveiling the decrease of the overall photorefractive effect contrast. This result highly affirms that the Talbot-type-fringes are the dominating factor in the formation of the SBG.

**Figure 12:**
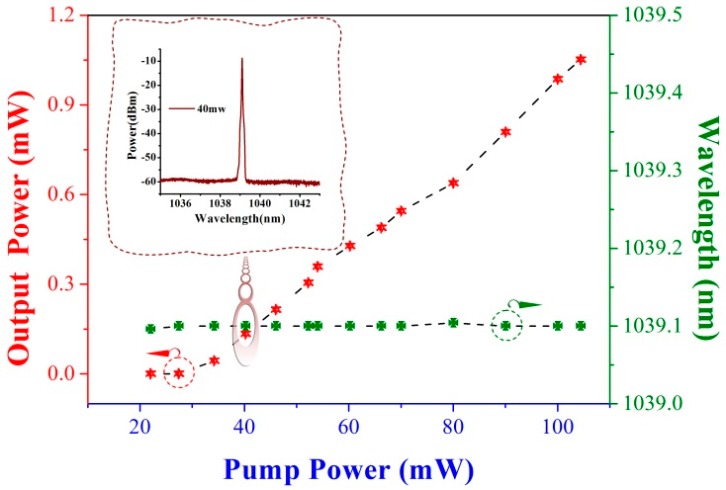
Changes of the power and wavelength of the laser versus pump power. Inset: the laser spectrum at pump power of 40 mW.

As a sequence, those gratings can be applied as Bragg reflectors operating at 1-μm-wavelength, taking advantage of the high reflection of the third harmonic resonance [30]. We fabricate two wavelength-matched gratings in a photosensitive fiber and then splice them to a 20 mm length of ytterbium (Yb)-doped single-mode active fiber to form a Fabry-Perot cavity with an interference spectrum in a 1-μm-wavelength window. The Yb-doped fiber has core and cladding diameters of ~3.5 μm and 125 μm, respectively. The numerical aperture (NA) is 0.18. The numerical aperture (NA) is 0.18. By connecting a 980 nm pump laser, the DBR laser operated at a wavelength of 1039 nm can be formed by virtue of the strong reflectors and the high pump absorption of 1200 dB/m in the Yb-doped fiber. By tuning the 980 nm pump laser from 0 to 100 mW, the output characteristics of the third harmonic DBR laser can be measured, which is shown in Figure 12. The laser presents a positive response to the pump power, whose pump threshold is 20 mW. At pump power of 100 mW, the laser can output power over 1 mW. During the pump-tuning period, the laser wavelength stably stays at 1039.1 nm, due to the lower pump absorption of the grating region (5 dB/m).

**Figure 13:**
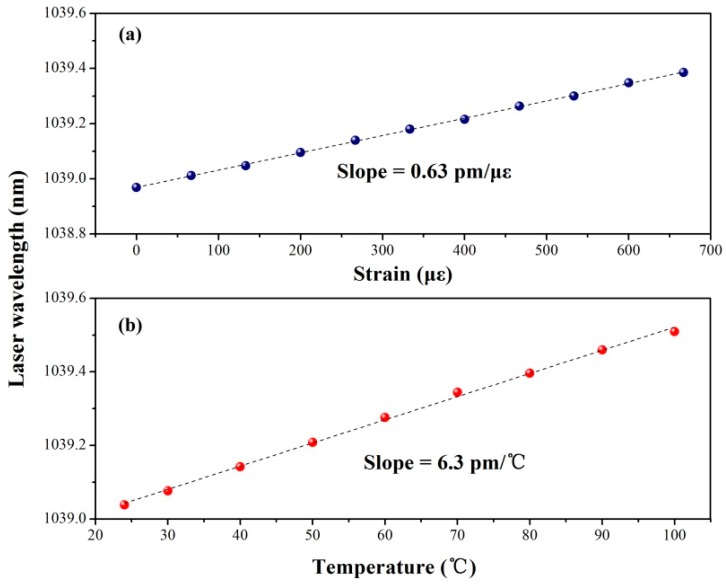
Laser sensitivity of the wavelength responding to the (**a**) strain or (**b**) temperature.

Maintaining the pump power at 40 mW, the laser is used in the following study, whose spectrum is displayed in the inset of Figure 12. At first, the strain response of the third harmonic DBR laser is measured by loading an axial tension onto both sides. By increasing the tension gradually, the correlation between the strain and laser wavelength can be obtained. According to Figure 13a, the strain sensitivity is 0.63 pm/με. Then the temperature response of the laser is tested. From room temperature to 100 °C, the correlation between the temperature and laser wavelength is shown in Figure 13b. The temperature sensitivity is 6.3 pm/°C. The results are highly coincident with the sensing characteristics of the third harmonic gratings [17]. The sensitivities corresponding to the temperature and strain are significantly lower than those of 1550 nm DBR laser [31]. Therefore, the third harmonic DBR fiber laser can present higher stability in wavelength depending on the lower disturbance influenced by the strain and temperature.

**Figure 14:**
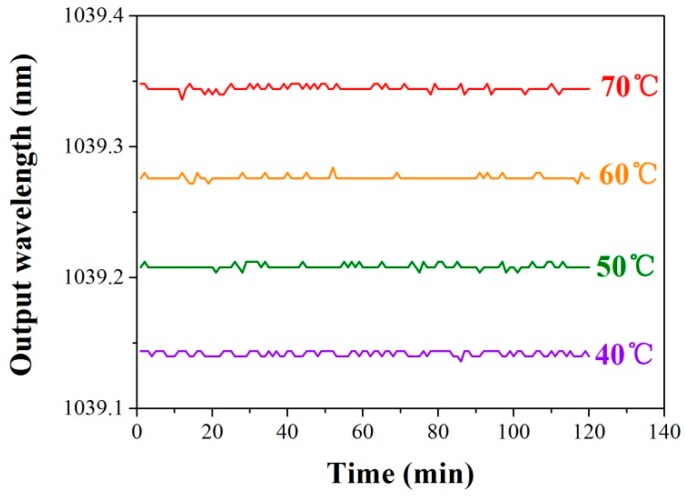
Long-term stability of the lasing wavelength at different temperatures.

The long term wavelength-stability of the laser is tested at different temperatures. From Figure 14, the wavelength variation is less than 2 × 10^−6^ nm at each temperature over two hours. As a sequence, a wavelength-reliable DBR laser operating at 1 μm can be presented providing the potential in optical lattice clocks, space probes, and sensors [32,33,34,35].

Furthermore, an extensional thought should emphasize on the Talbot-type-fringes. Compared with other frequently used inscription laser with wavelengths of 244 nm or 248 nm, the 193 nm laser not only provides higher index modulation efficiency without hydrogen-loading but also supports a larger Talbot length. Thus, a single-layer modulation structure in the fiber core can be realized with greater possibility, which is important to form higher order harmonic reflection in the grating. It can be estimated that phase mask with pitch as low as 1044 nm, which results in a Bragg wavelength of 1513 nm, can still guarantee the Talbot length larger than 6 μm, twice the fiber core that we used. Therefore, the phase masks designed for 1.5 μm waveband gratings can be utilized in fabricating third harmonic lasers operating at the wavelength within the gain spectrum of the Yb-doped fibers. Thus, the secondary grating formation mechanism offers great flexibility for designing the wavelength of the fiber lasers.

## 4. Conclusions

In this paper, a unique grating named the secondary Bragg grating is presented. Depending on the Talbot-type-fringes of the inscription light, two interleaving modulation structures would be formed along the fiber axis. A large contrast of the index change exists as if the diameter of the photosensitive core is equal to half of the Talbot length. By a large amount of the cumulative fluence, which is needed in Type-IIa FBG inscription, the weaker periodical modulation would result in the generating of the SBG. Sensing applications of the DBR fiber lasers based on those SBGs are demonstrated. For instance, a DBR fiber laser with ultra-short dimension can act as a compact temperature sensor, even in extreme temperature environments; a threshold-tunable DBR fiber laser can be utilized as the flexibly-managed high-temperature alarm; the third harmonic reflector-based fiber laser presents an anti-jamming 1-μm wavelength output, providing the potential in space probing and coherent optical detection. Given that the investigation of this new type of grating is still underway, the future results may further enrich the study and the application development of the FBG devices.
